# Why do Patients Forget to Take Immunosuppression Medications and Miss Appointments: Can a Mobile Phone App Help?

**DOI:** 10.2196/publichealth.5285

**Published:** 2016-04-04

**Authors:** Ajay Israni, Carl Dean, Brian Kasel, Lisa Berndt, Winston Wildebush, C Jason Wang

**Affiliations:** ^1^ Hennepin County Medical Center Department of Medicine Minneapolis, MN United States; ^2^ University of Minnesota Department of Epidemiology and community Health Minneapolis, MN United States; ^3^ Minneapolis Medical Research Foundation Minneapolis, MN United States; ^4^ University of Minnesota Department of Renal Diseases and Hypertension Minneapolis, MN United States; ^5^ Center for Policy, Outcomes and Prevention and Division of General Pediatrics Stanford University School of Medicine Palo Alto, CA United States

**Keywords:** adherence, immunosuppressive medications, appointments, mobile phone

## Abstract

**Background:**

Kidney transplant recipients must adhere to their immunosuppressive medication regimen. However, non-adherence remains a major problem.

**Objective:**

The aim of this paper is to determine how kidney transplant recipients remember to take their medications, and assess their perception and beliefs about adherence to immunosuppressive medications and barriers to medication adherence. In addition, we aim to assess perception and beliefs about willingness to use a hypothetical, mobile phone app to improve adherence.

**Methods:**

We conducted a qualitative study that included an average of three home or workplace visits of kidney transplant recipients (N=16) from a single urban transplant center.

**Results:**

The qualitative study revealed that transplant recipients understood the importance of taking their immunosuppressive medications and this motivated them to take their medications. The visits showed that most participants have incorporated medication use into their daily lives and that any minor deviation from daily routines could result in non-adherence. Participants also reported other barriers to adherence. All participants were interested in using an app to remind them to take their medication; however, they reported potential barriers to using the app.

**Conclusions:**

Although kidney transplant recipients understood the importance of medication adherence, there were significant barriers to maintaining adherence. Participants also reported interest in using a mobile phone app.

## Introduction

Despite advances in solid organ transplantation, allograft failure due to non-adherence to immunosuppressive medications and follow-up visits remains a major problem [1-12]. Several clinical studies in kidney transplantation have highlighted the negative impact of non-adherence on kidney function post-transplant [2-8]. Post-transplant care requires at least two or three oral immunosuppressive medications and intensive monitoring of kidney function during frequent follow-up visits [1]. Therefore, there is a dire need to understand how patients take their immunosuppressive medications.

A novel approach to improving adherence is the utilization of mobile phone medication adherence apps and the use of text messaging (short message service, SMS). A national study by the Pew Research Center reported that 90% of all American adults own a mobile phone, 58% own a mobile phone with app capabilities, and 42% own a tablet computer [13]. The use of mobile phones is increasing among kidney transplant recipients, especially among African Americans and those with lower household incomes [14]. This technology has shown promising results of improved medication adherence in the areas of HIV, tuberculosis, diabetes mellitus, asthma, and emergency department-prescribed antibiotics [15-20]. Adherence apps linked to an electronic medication tray and a wireless Bluetooth-enabled blood pressure monitor has been tested in kidney transplant recipients [14,21]. To the best of our knowledge, no other adherence apps tailored to kidney transplantation have been tested.

In order to understand kidney transplant recipients’ perceptions and beliefs regarding barriers to medication adherence and potential use of a mobile device platform (presented as paper mock-ups) to improve adherence, we conducted one-on-one semi-structured interviews and home or workplace visits among kidney transplant recipients.

## Methods

### Participants

Transplant recipients were enrolled from August to December 2012 at Hennepin County Medical Center (HCMC). The purposeful sample of ethnically diverse transplant recipients was enrolled based on the perceptions of nurse coordinators that the subjects were either highly adherent or poorly adherent to immunosuppressive medications. Inclusion criteria included the ability to communicate in English, and residence in the Twin Cities area and surrounding suburbs in order to improve feasibility of conducting the interviews. At HCMC, among the 226 recipients transplanted from 2010-2012, 18.1% (41/226) were African American and 3.1% (7/226) were Hispanic. The transplant center actively follows approximately 800 recipients with a kidney transplant. The post-transplant care at the transplant center is routinely managed by a nephrologist. All transplant recipients are seen by a transplant pharmacist at a minimum interval of 2 weeks, 4 weeks, 3 months, 6 months, 12 months, 18 months, and 24 months post-transplant, and yearly thereafter.

All participants were invited for one-on-one semi-structured interviews and home or workplace visits. Initially, twenty participants were enrolled. Four participants dropped out for various reasons such as depression, living with boyfriend, construction at home or traveling. The data on these participants are not included. Each participant gave informed consent and the study was approved by the Human Subjects Research Committee at HCMC.

### Procedures

Guides for the semi-structured interviews and home or workplace visits were developed with input from the study investigators. The questions used in the guide, which include closed and open-ended questions, are shown in Textbox 1.

The interviews and visits were conducted by the same individual (BK) who was coached on anthropologic approaches by CJW and/or AI before and weekly during the study. Key findings were discussed with other investigators (CJW, AI) in weekly to bi-weekly meetings for reflection. No clinic or medical personnel were present during these encounters to ensure solid rapport and full disclosure by study participants. The study design included individual interviews for several reasons such as (1) the interviews were conducted during several home or workplace visits; (2) they increased the interviewer’s familiarity with study participants’ perceptions and beliefs; and (3) they improved trust-building efforts to elicit sensitive data about adherence to medications and appointments during home or workplace visits.

The semi-structured interview methodology was informed by inductive reasoning. While quantitative studies use statistics to establish reliability and validity, qualitative research relies on trust and rapport-building. The semi-structured interviews require the skill of keeping participants on task, maintaining rapport with all to ensure equitable but not peer-pressured participation, and maintaining a high degree of comfort discussing sensitive topics [22]. All interviews were audio taped and reviewed by two authors (BK, AI). The later portion of the semi-structured interview consisted of an oral survey to collect demographic and transplant-related information. The participants’ electronic medical records were reviewed for other medical information.

**Kidney transplant recipients’ interview questions from the Moderator’s Guide. Question number 16 and onwards were designed to assess perception, beliefs, and willingness to use a mobile phone app (presented as a paper mock-up) to improve medication adherence.**

Question

1. Why do you choose to take your immunosuppression medications (eg, prednisone, Gengraf, Cellcept)
2. What gets in the way of taking your immunosuppression medications?
  1. Does anything ever prevent you from remembering to take your medications?
  2. What happened last time you did not take your immunosuppression medications?
3. Have you ever chosen to miss a dose when you were short on money?
4. Have you ever chosen to miss a dose when you went out of town?
5. Have you ever chosen to miss a dose when you felt depressed?
6. What pharmacy or pharmacies do you use to get your immunosuppression medications?
7. What don’t you like about your pharmacy’s service for immunosuppression medications?
  1. Why did you run out of your immunosuppression medications?
  2. How many days were you out?
  3. What can be done to try and prevent this in the future?
8. What do you like about your pharmacy’s service for immunosuppression medications?
9. Do you have a keychain or travel pill holder?
  1. When do you usually use it?
10. Have you ever had any undesired side effects from your immunosuppression medications?
11. Have you ever chosen to miss a dose when you think your medications may have unwanted side effects?
12. In last three months, has any emotional or physical suffering prevented you from taking your medications?
13. Why do you choose to go to your doctor and lab appointments?
14. What gets in the way of making it to your doctor and lab appointments?
  1. What gets in the way of remembering?
  2. What happened last time you did not make it to your doctor or lab appointment?
15. What happened to make you forget your appointment?
16. Would you be interested in getting reminders for lab and doctor appointments?
  1. When would you like to get these reminders?
17. Are there certain situations where you do not want to get reminders for doctor and lab appointments?
18. Would you be interested in getting reminders for taking medications every day?
  1. Would you be interested in getting reminders for taking medications only if you forget?
19. Are there certain situations when you do not want to get reminders for medications?
  1. How often would this happen?
20. Would your social life ever get in the way of getting a reminder?
21. Would your work life get in the way of getting reminders?
  1. Would you be ok with a vibrating reminder going off during a meeting and/or quiet event?
  2. Would you be willing to take a mobile phone picture of your medications to prevent the reminder alert?
22. Would it be okay to have words like "kidney transplant" or "medications" show up on reminders on your phone?
  1. Are you comfortable with your friends, family members, and coworkers knowing that you have a kidney transplant?
  2. What about random people around you?

### Data Analysis

Qualitative data was collected and analyzed according to the principles of grounded theory. Coding is an iterative process produced through several discussions by researchers to establish broad codes and then further refine the code.

The digital audio tapes of the interviews were transcribed verbatim and visit notes were transcribed to produce 124 pages of text. Coding was done manually without the use of specialized coding software. Two authors (BK, AI) reviewed the transcripts manually, line-by-line to generate a preliminary coding scheme through consensus: a list of codes and corresponding definitions. Therefore, no formal measure of agreement was generated for the codes generated by the two authors. The codes were ordered by thematic similarity or relationship into a project codebook. The resulting themes reflected ideas that emerged over a variety of areas of inquiry by the research team. Quotations that most closely represented the theme were then selected to support the analysis. The data from the transcripts was supplemented with notes taken during home or workplace visits.

## Results

The characteristics of the study participants are shown in Table 1. All subjects were interviewed at their residence; however one subject was interviewed at his place of work and one at a coffee shop near her place of residence. The average of 3 visits per participant showed that most participants have incorporated medication use into their daily lives, such as one participant keeping morning medications by the car keys in order to remember to take the medications before leaving for work in the morning. However, any minor deviation from daily routines may result in non-adherence. With one exception, no participants had used electronic reminders on a phone (not a mobile phone) to alert them to take their medications. The one subject that did use reminders utilized the alarm feature on his mobile phone and would not turn off the alarm until he took his medications. None of the participants had mobile phones with app capabilities.

With one exception, all participants used pill boxes due to the large number of medications required. Some subjects also had a smaller pill holder, such as a plastic bag, to carry a single dose of medications. Having this smaller pill holder sometimes resulted in missed medications as some subjects forgot to put medications in this smaller pill holder or forgot to ingest the medications due to deviations from their daily routines.

Seven families of codes or themes were identified and yielded 133 non-discrete quotations. These seven main themes structure the rest of this paper.

### Motivated to Take Immunosuppression Medication

Study participants demonstrated a clear understanding of the negative consequences of not taking their immunosuppressive medications (Theme 1, Table 2). They mentioned that non-adherence could increase their risk of rejection and increase risk of returning to dialysis. Taking the medications reassured them they would not experience rejection.

A participant described his motivation for taking his immunosuppression medications: "So I don’t have to go back on dialysis." Participants dreaded the return to dialysis. One participant remarked: "...I want this kidney to work. I don’t like the alternative (dialysis)."

### Participants' Perceived Barriers to Taking Immunosuppression Medications

The participants reported barriers to adherence such as forgetting to take or renew medications, procrastination, being short on money, having depression, distractions, change in daily routine, forgetting medications while away from home, alcohol use, and falling asleep before taking evening dose (Theme 2, Table 3)

"I thought that I took it that morning, but then later on in the afternoon when I look at the medication package I found out it was not taken." Some patients found it difficult to maintain compliance while traveling:

If I’m in California I have to get up at 5:30 and take my (morning) medications at 6:00…I take them at 8:00 central time regardless of what time zone I’m on.

Other participants admitted to simply overlooking their medications "…I just had forgotten to renew them…"

### Reported Factors That Helped Participants to Take Their Immunosuppression Medications

Factors that help patients remember to take their immunosuppressive medications include creating a routine around taking their medications, monitoring their medication supplies regularly, using a travel-sized pill holder, having a support system, making medications a top priority, taking medications earlier in the day and trusting their physician (Theme 3, Table 4). Surprisingly, only one participant set an alarm.

I kinda like my alarm (that just) goes off. It only goes off twice a day. So once in the morning, I have it go off at 8:30 so that if I’m asleep then I can get up and be ready by 9:00 o’clock to take my meds. And then at night it goes off by 8:50 so I can take my meds at 9:00. It gives me a little 10 minute window

Another participant noted the help he received from the pharmacy:

The pharmacy has been doing well. They remind me about my medication. Call me to check on my medications to know which one I should…refill…So they are a help to me.

### Motivated to Keep Appointments

The study participants were motivated to keep their appointments. They referenced their appointments as a way to stay alive and healthy and to monitor their kidney function (Theme 4, Table 5).

Well I choose to go to my appointments so we can monitor the kidney function. Make sure all my levels are where they need to be.

Another participant cited similar curiosity to their health status as a reason to keep appointments:

It’s a necessary part of my treatment. I want to know. I’m curious to see how I’m doing. I like to talk to (the doctors), I have questions, I’m curious.

### Participants' Perceived Barriers and Motivation to Keeping Appointments

The study participants reported some barriers to keeping appointments such as transportation, busy schedule, forgetting, not feeling ill or due to personal sickness or sickness in the family (Theme 6, Table 6).

My schedule. I mean I have to do a lot of working around my schedule and then sometimes the clinic is not (open), I think the clinic needs to have extended hours because it just makes it hard

Some patients found it difficult to maintain appointments when they were not feeling well:

I think I’m alright because I’m not that bad right now. So I didn’t go…

### Reported Factors That Helped Participants to Keep Appointments

The participants reported scheduling recurrent visits on the same day of the week, various types of reminders and support from others as strategies to keep appointments (Theme 6, Table 7).

"When they first started it (the appointment) was Monday, Wednesday, Friday, Monday, Wednesday, Friday then it went down to just Wednesdays, and now I’m going to (every) two weeks so it’s every other Monday." Some participants utilized electronic medical record patient communication tools to maintain appointments:

I have MyChart and I get emails…‘You have so and so many appointments,’ and I’ll click them and then oh yeah that’s a reminder.

### Perceived Barriers to a Medication Adherence App

The participants voiced concern regarding their mobile phone being turned off or inaccessible, missing the alarm, and inflexibility of the app to adjust to irregular schedules (Theme 7, Table 8).

If I was in a job interview I would have my phone possibly off or on vibrate…in a meeting it is just common courtesy to do that (turn off phone)…Anywhere from one hour to all day. But there are frequent breaks and during that time everyone checks their phones.

Other participants expressed concern about ignoring their phone during the day: "…when you are pretty busy or when you are enjoying sometimes you don’t look at the phone."

**Table 1:** Characteristics of study participants (N=16).

Characteristic		n (%)
Recipient male		10 (63)
Recipient race		
	Asian	2 (13)
	Caucasian	9 (56)
	Hispanic	1 (6)
Income		
	<$15,000	5 (31)
	$15,001-$30,000	3 (19)
	$30,001-$45,000	1 (6)
	$45,001-$60,000	1 (6)
	$60,001-$75,000	2 (13)
	>$75,000	4 (25)
Employment		
	Employed full-time	4 (25)
	Employed part-time	2 (13)
	Unemployed	7 (44)
	Retired	1 (6)
	Full-time homemaker	1 (6)
	Student	1 (6)
	Unable to work	0
Primary insurance		
	Private	10 (63)
	Medicare	5 (31)
	Medicaid	0
	Other	1 (6)
Had secondary insurance		9 (56)
Years of schooling, mean (SD)		13.6 (3.2)
Marital status		
	Married	9 (56)
	Separated	1 (6)
	Single/never married	2 (13)
	Divorced	3 (19)
	Living with someone	1 (6)
Transportation		
	Own a car/family owns a car	14 (88)
	Public transportation	1 (6)
	Other	1 (6)
Cause for end-stage renal disease (ESRD)		
	Diabetes	1 (6)
	Hypertension	5 (31)
	Polycystic disease	1 (6)
	Glomerular	2 (13)
	Other	7 (44)
Duration since transplant in years, mean (SD)		4.2 (3.9)
Self-described health status		
	Excellent	0
	Very Good	6 (38)
	Good	7 (44)
	Fair	3 (19)
	Poor	0
Living donor		12 (75)

**Table 2:** Motivated to take immunosuppression medication (coded 15 times).

Authors’ interpretation of interviews	Supportive quotations from patients	Study identification number
To avoid dialysis	"Because I want to keep my kidney working right. The alternative is dialysis, and that’s not fun."	114
Because it was prescribed by the doctor	"For myself I never choose medications because I don’t know anything medical so whatever they recommended I eat it. So it’s not my choice at all."	116
To stay alive	"I take it (medication) because it helps me to continue to live to see another day, and also keeps me from going into the hospital…If I don’t take it I’m only hurting myself."	108
To prevent rejection	"I don’t want to have my kidney reject."	106
To be adherent because of a death in the family due to non-adherent behavior	"It’s amazing what missing your medications can do to you, and my father is a good example of that. He (my father) might have been able to prevent that stroke had he taken his medications that morning. He felt good, he felt like he was not in a lot of danger."	108

**Table 3:** Perceived barriers to taking immunosuppression medications (coded 35 times).

Authors’ interpretation of interviews	Supportive quotations from patients	Study identification number
Forgetting to bring meds to work and work schedule is inflexible	"Right now my main thing is my work schedule… I forgot to bring it along (to work) in a plastic bag in my pocket… I don’t have enough time to go back and get them; otherwise it could be a termination from one of the jobs."	114
Procrastinating at taking meds	"Well so I take so many pills like in the morning I think there are fifteen or sixteen and at a point you get so tired of taking them and you say or you think ‘I’ll take them a little later’. Then you get to doing things and then something else comes up and then you don’t."	113
Procrastinating at ordering meds	"I procrastinate about ordering my meds. Fairview (pharmacy) will call me and then I’m busy doing something else or in the car or something where they say can you look at your meds now and I say no I can’t if I’m driving around."	115
Patients cannot feel the harm they are inflicting on their kidney	"If you are feeling very good and enjoying things then you forget you are sick. Easy to miss it…"	116
Due to being short on money	"I had run out (of money) in the middle of pay days. Even now I try to schedule it, like my wife gets payday Friday and then she and I both get our retirement on the first of the month…I have missed my immunosuppression meds for a day. There was another pill, it was really expensive."	113
Due to being depressed	"There was a time when I’m sure I was depressed…It was a duration (of non-adherence) probably of two days max I’m thinking…You just get that lethargic feeling, ‘I’m sick of these pills,’…You take them late but you try not to miss them completely."	113
Pharmacy did not supply meds on time	"About one or two times it was delayed because of the…mail…I was called by the clinic, ‘We are sorry we will be late because they were not picked up but by tomorrow it will be around,’ and surely I receive it in that time. I ran out of it but I got it the day or two after.'"	101
Patient forgot to take it due to a distraction	"It was a night, I’ve never forgotten to take the morning pills, it’s always been the night pills…I will sometimes get caught up in doing something else and my short term memory can sometimes be bad so sometimes. If I know I don’t have to take this until 10:00 o'clock I get busy doing something and then before I know it it’s the next morning."	106
Patient didn't want to take meds with alcohol	"I think maybe one time because I was enjoying myself too much. And I was like I don’t think I should be taking my pills after just consuming alcohol. So I just didn’t take it. Yeah I was out partying and enjoying myself too much.	110
Patient forgot to renew his/her prescription	"No, well not deliberately anyway. I just had forgotten to renew them…"	119
Patient changed pharmacies (due to insurance coverage)	“They (pharmacy) have just had issues with the exact company (insurance) that I need to use mainly because the companies (insurance) don’t understand how to use Medicare as a secondary insurer… So there has just been an issue mainly with finding a place that will bill Medicare. That is the issue….I would be on the phone with the insurance company and I would beg, and I would say, ‘I will get sick without them’…It was just kind of a mess. It was mainly on the administrative side."	106
Medications were stolen	"The one time I was living in the other place I was living in, they delivered them and left them at the door because it’s a safe neighborhood and they weren’t there when I got home from work…They said they delivered them, they traced it to the door and one of my roommates…so now I get them delivered to me at work and everyone leaves them alone when I’m not there."	119
Patient forgot to take their meds when they were out of their house	"I was late. It was just the first time I was off my schedule…I was at somebody else’s house and we were talking at a dinner party or something…"	107
Patient fell asleep and missed PM meds	"Oh yeah, like I said before I had forgotten once or twice in the evening. Usually it’s the evening dose. It’s only a couple of pills and I fall asleep and then oops, ya know. And it’s not like I deliberately don’t take them."	119
Taking meds at different time than usual because the patient has their trough levels checked in the lab	"The morning of the Prograf (trough level) draws, you can’t take your Prograf so after the appointment you have to remember to take Prograf because I didn’t take it with my other pills. So one particular day I was…distracted so when I left the clinic I was not thinking and then four or five o’clock came and I took my pill box out of my purse and said, ‘What are those two Prograf still doing there, oh shoot I didn’t take it.'"	110

**Table 4:** Helps patients to take immunosuppression medications (coded 23 times).

Authors’ interpretation of interviews	Supportive quotations from patients	Study identification number
Everyday bi-daily alarms	"Because you might be doing something, you might be driving and then if you turn it off once you stop driving you forget so I don’t turn it off until I take the meds…It snoozes automatically for five minutes."	100
Taking pills around the same time and creating a routine	"I have a system and…my system it’s (medications) the last thing I do before I walk out the door. And the way I know I have to take my medication is because it’s by my keys. Keys to drive the car, keys to lock the door, keys to get into my office. So when I get up and do all these things that I need to do to get prepared to go out, the last thing I see is my medication by my keys…I have ten tubes of medications. I load all those up at the start of the week."	108
Patient monitors med supplies regularly	"Every weekend I check my medication. Because I do the packing in the separate (pill-boxes). So when I find out this is getting short…I call them and tell them those medications that I need and she told me in a day or two they will be here."	101
Patient uses a travel pill holder	"So if I’m going to go somewhere and I know I will be gone, I got a little pill box, so I pop them out and put them in a pill box. So say I’m at Bible study and we don’t get out until 9:30 and I take my meds at 9:00. My alarm will go off, pop them pills."	100
	"Just a small sandwich size Zip Lock for the one dose that I’m taking. Like in the morning if I have to work AM. And everybody knows I just say, 'I’m going to take my meds, and they go, ‘Okay.'"	105
Patient has a support system	"Right now I keep a very strict regimen of when I take my meds and like I said even my husband (helps)… (he) was like ‘do you have your meds with?’…The support system I have (really helps). So he is my reminder too."	102
Patient makes taking medications a top priority	"…I make this a top priority…My meds come first."	102
Taking medications at times that are easy to remember	"I like to take my pills sometime between 7:30 8:00 o’clock in the morning and 7:30-8:00 o’clock at night. 7:30 is a time when I look at the clock at night because this is when my son has to come in... Boom pills go along with that."	112
Patient trusts their doctor	"I trust the doctors a little bit too much to be doing that (not taking medications)."	114

**Table 5:** Primary motivation to keep appointments (coded 16 times).

Authors’ interpretation of interviews	Supportive quotations from patients	Study identification number
To stay healthy	"Because when my hemoglobin is very low then I feel very weak and quick tired. Sometimes when I go (to the clinic) the nurse is like, ‘Only seven percent of blood is there.’ It’s very low."	116
The patient likes and believes in her doctors care	"…and I appreciate that about them because they are so vigilant. So I wouldn’t not go to them."	105

**Table 6:** Perceived barriers to keeping appointments (coded 13 times).

Authors’ interpretation of interviews	Supportive quotations from patients	Study identification number
Being distracted by kids	"…I don’t really have anyone to watch my kids so I may have to bring them with me which I’ve never done and I don’t really want to do that because it’s very distracting and hard to do with two of them."	106
Not having transportation	"… Mostly I contact the transport company then sometime (they are) a little bit late. Sometime (they are) very early that I have to go and wait for almost one hour before I can see my doctor. Sometime they are late that when I reach there the doctor and nurses say we will consider seeing you but you are late."	101
Due to double booking doctors’ appointments	"It’s usually been a scheduling conflict…I would schedule a lab appointment down here and something at (hospital) at the same time."	114
Sickness	"Nothing (gets in the way of my appointments) unless I’m really sick with the flu or something."	119
Reminder too far in advance	"I guess if it’s (the reminder) too far in advance then it’s not really that meaningful for the same reason I sometime forget to take (my medications), if it’s too far in advance it’s not really that immediate. I’m really more in the moment."	106
Oversleeping	"Once I missed and it was just that I overslept."	113
Forgot the appointment	"…I did (forget), but I thought it was the following week. I think we got our dates switched…"	119

**Table 7:** Helps patients to keep appointments (coded 22 times).

Authors’ interpretation of interviews	Supportive quotations from patients	Study identification number
Visits scheduled on the same day of the week or month	"Just mainly towards the end of the month so I remember that it’s getting towards the end of the month so I should see when the next lab is."	115
Patient puts transplant clinic appointment card in his/her wallet	"The card...I put it in my wallet in the front."	117
Phone call reminder from the doctor’s office	"Well it gives me a chance to get to know I’m on someone’s mind as far as the clinic. I get a chance to hear a nurse’s voice on the phone and kid around with them on the phone. So receiving that call does a lot. It shows that they’re on their job making sure that patients don’t forget their appointment. And you kind of build a little rapport with them on the phone. And I kind of look forward to those calls."	108
Work is accommodating and flexible	"…I have to go in (to a clinic appointment) on Friday and I’ve already emailed my boss saying that I will be working from home because I have a doctor’s appointment."	102
Making appointments is a top priority	"…I make this a top priority. This is a very important priority that we keep in the back of our minds at all time. My meds come first. Doctors’ appointments are first."	102
Patient has written personal reminders	"…I will write myself a note to remember. For example, ‘I actually have to schedule one for December, but I know it is important enough that I actually have to write myself a note to remember because if I wait until December, chances are they just get really busy, so I need to call in advance."	106
Email doctor through MyChart	"Yup, and that way it’s easier to remember because it’s (email) in writing. Then I can always go back on MyChart and say, ‘Hey that’s what they said,’ so I can always remember."	114
Mobile phone calendar reminders for appointments	"…my medical appointments I have set up for both a day and a half an hour before I’m supposed to go. So I get a one day alarm and I get a thirty-minute alarm. But once I get that one day alarm, it’s already in my mind that I have to go tomorrow. Then I have thirty minutes, okay time to get ready to go."	108
Mobile phone calendar reminders for appointments	"Yeah, I usually have it set for forty-five minutes before hand so I have time to get there and stuff." (BL) "And is the same thing for lab appointments?" "Yeah, mmhmm."	119

**Table 8:** Future perceived barriers to the app (coded 9 times).

Authors’ interpretation of interviews	Supportive quotations from patients	Study identification number
Phone turned off periodically	"I will turn it on silent at night, but I don’t shut it off."	106
App alarms, though helpful, may become too annoying so the patient turns them off	"Well like for Prograf (trough levels)…they call you the day before and remind you to take your Prograf at a certain time. And just because I’ve been doing it for over a year it’s like I know that I have to take it. Even though it is good sometimes because I have (forgotten)."	110
	"Straight reminder that’s maybe after a while going to be a nuisance if you get the reminder….after you’ve already taken them and then you get something that says, ‘Take your meds!'"	113
People not having their mobile phone on them all the time	"I have a little spot on the counter and so it’s plugged in there right now and if I were to go out and do something in the garage it wouldn’t be with me. My son comes home and we go out and play, it’s not with me."	112
If the app is not flexible enough to work with irregular schedules	"So like I said send me a message before I go…If I work tomorrow at 6:00 then an automated (message) at 5:30 saying do you have your medications ready for work or something."	114
It may cumbersome to take a mobile phone picture to document medication ingestion to prevent the app alarm. (This option for the app was posed to the participants)	"That’s…easy to forget to take the picture. And then they are going to think that I don’t take my medication..."	117

## Discussion

### Principal Findings

The kidney transplant recipients that participated in these interviews demonstrated an understanding of the importance of immunosuppression medications. Commonly cited reasons to remain compliant included to avoid dialysis, stay alive, prevent rejection, and witnessed complications from noncompliance in family members (Table 2). Despite these appropriate concerns, participants shared numerous perceived barriers to maintain perfect adherence to immunosuppression medications. These barriers included simply forgetting to take or renew medications, procrastinating, being short on money, having depression, getting distracted, change in daily routine, forgetting medications while away from home, alcohol use, and falling asleep before taking evening dose (Table 2).

We found it reassuring that our participants identified understanding the importance of immunosuppression medication. In a previous publication using mailed surveys, Chisholm-Burns et al identified that kidney transplant recipients were more likely to miss doses of medications when they acknowledged a weaker belief on the necessity of maintenance immunosuppression medications [23]. Our results suggest that among the participants in our study, present efforts to educate kidney transplant recipients on the importance of immunosuppression are successful to a large extent and should be continued [24,25].

Similar to our findings, several previous investigations have reported patient-identified barriers to medication compliance. Consistent with our findings, these publications have cited limited financial resources, and forgetfulness, interruption of daily routine, travel or leaving home, change in dose, run out of medications, and no immediate access to a pharmacy as barriers to compliance [23,25-27]. Intentional non-adherence has been found previously to be significantly lower than unintentional non-adherence among kidney transplant recipients [26,28]. We found a similar trend among responses in our study. This suggests that larger improvements in adherence may be seen with strategies designed to remind patients to take medications and facilitate delivery and administration of medications in addition to education them on their importance. The behavioral change theories commonly used in mobile app applications, such as health belief model (perceived susceptibility, perceived severity, perceived benefits, perceived barriers, cues to action, and self efficacy), and social cognitive theory (self efficacy, expectations, behavioral capability, observational learning, reinforcements, and reciprocal determinism), combined with behavioral techniques such as nudges (eg, loss aversion, anchoring, and benchmarking), can facilitate medication adherence [29-31].

Interestingly, when we tested the idea of using a paper mock-up of a mobile phone app specifically designed for kidney transplant recipients we receive generally positive responses. While some participants reported interest in this utility, others raised concerns with this proposal such as what would happen when the phone was switched off or not in their immediate proximity, developing annoyance to a phone alarm, and the inflexibility of the app to adjust to chaotic and changing schedules were concerns raised by participants (Table 8). A recent review of mobile phone medication adherence apps identified 160 currently on the market. While their efficacy remains untested, medication adherence apps offer many potential benefits that include constant accessibility, ease of use, low cost, and ability to consolidate all medication-specific information for the patient and provider. Based on their respective capabilities and ease of use, the review distinguished ten medication apps as noteworthy. These apps are MyMedSchedule, MyMeds, MedSimple, Med Agenda, RxmindMe Prescription, Dosecast, TRxC (Beta) MediMemory, PillManager, and MedsIQ Individual/Multi-user. No free-standing app, however, was specifically tailored to kidney transplant recipients [32].

In response to the concerns identified by participants in this study, it is our belief that a mobile phone app designed for kidney transplant recipients would have the following features. First, it would be easy to input a multi-drug regimen into the app. Second, the app would offer a choice of options for notification of medication due time. These options would include a standard text message, an email generation or alarm feature when a medication would be due. Third, the app could generate a reminder of a due medication without cellular connectivity. Fourth, the app would feature a "snooze" button to allow for an additional reminder to take the medication if the user was unable to complete the task immediately. Fifth, instead of just a simple reminder, the app would have a feature that allow the user to voluntarily indicate if they did or did not take the medication and note the time when this occurred. This data could be collated and downloaded at the physician office as a way to prompt discussion between the caregivers and the patient of methods to improve adherence. Sixth, the app could communicate with the patient’s primary pharmacy and facilitate refills as they become necessary to prevent lapses in medication availability. Finally, any app created based on this input would have to be tested extensively among transplant patients to ensure the app engages the patients through social competition [33], and individual encouragement [34]. Previous focus groups have shown that peer-support relationships and talking to other patients on dialysis therapy have motivated transplant recipients [33].

To our knowledge, no studies utilizing mobile phone apps to improve outcomes in kidney transplant recipients have been published. One study of pediatric liver transplant recipients showed significant reduction in variation of tacrolimus levels using text messaging reminders and suggests that adopting this reminder system may lead to improved medication adherence and decreased episodes of rejection [35].

### Limitations

There are several limitations in the current study. First, although we had a diverse group of participants based on their socio-economic information, this study was conducted with a small sample size of prevalent patients in a single center study. In addition, common themes were appearing in the qualitative data provided by the participants. Second, four patients with unstable living situations or depression dropped out of the study; thus, it is possible we may have missed some issues specific to vulnerable populations. We did not include patients that lost their allograft due to noncompliance. However, our findings are generally consistent with previous survey responses regarding the reported barriers to medication adherence from other transplant centers [23,25-27]. None of our subjects had mobile phones, despite no purposeful sampling in regards to access to mobile phone was used. The mobile phone use in 2012 at our county hospital was likely low. Lastly, we did not measure adherence in our study participants; thus, the barriers and facilitators to adherence may be participants’ perceptions.

From this qualitative study of medication adherence practices, it is clear that patients have incorporated medications into their daily lives and schedules, but that any variation to this schedule increases the risk of noncompliance. In addition, there are numerous barriers to medication adherence that participants in our studied identified, and participants utilized a variety of techniques to reduce the influence of these barriers. While no transplant-specific app exists presently, an app tailored to the needs of a busy transplant recipient may improve medication adherence.

## Abbreviations

HCMC: Hennepin County Medical Center

## References

[1] Israni AK, Snyder JJ, Skeans MA, Tuomari AV, Maclean JR, Kasiske BL (2009). Who is caring for kidney transplant patients? Variation by region, transplant center, and patient characteristics. Am J Nephrol.

[2] Weng FL, Israni AK, Joffe MM, Hoy T, Gaughan CA, Newman M, Abrams JD, Kamoun M, Rosas SE, Mange KC, Strom BL, Brayman KL, Feldman HI (2005). Race and electronically measured adherence to immunosuppressive medications after deceased donor renal transplantation. J Am Soc Nephrol.

[3] Butler JA, Roderick P, Mullee M, Mason JC, Peveler Rc (2004). Frequency and impact of nonadherence to immunosuppressants after renal transplantation: a systematic review. Transplantation.

[4] Kiley DJ, Lam CS, Pollak R (1993). A study of treatment compliance following kidney transplantation. Transplantation.

[5] Chisholm MA, Vollenweider LJ, Mulloy LL, Jagadeesan M, Wynn JJ, Rogers HE, Wade WE, DiPiro JT (2000). Renal transplant patient compliance with free immunosuppressive medications. Transplantation.

[6] Frazier PA, Davis-Ali SH, Dahl KE (1994). Correlates of noncompliance among renal transplant recipients. Clin Transplant.

[7] De Geest S, Borgermans L, Gemoets H, Abraham I, Vlaminck H, Evers G, Vanrenterghem Y (1995). Incidence, determinants, and consequences of subclinical noncompliance with immunosuppressive therapy in renal transplant recipients. Transplantation.

[8] Nevins TE, Kruse L, Skeans MA, Thomas W (2001). The natural history of azathioprine compliance after renal transplantation. Kidney Int.

[9] De Bleser L, Dobbels F, Berben L, Vanhaecke J, Verleden G, Nevens F, De GS (2011). The spectrum of nonadherence with medication in heart, liver, and lung transplant patients assessed in various ways. Transpl Int.

[10] Dew MA, Dimartini AF, De Vito Dabbs A, Zomak R, De Geest S, Dobbels F, Myaskovsky L, Switzer GE, Unruh M, Steel JL, Kormos RL, McCurry KR (2008). Adherence to the medical regimen during the first two years after lung transplantation. Transplantation.

[11] Kung M, Koschwanez HE, Painter L, Honeyman V, Broadbent E (2012). Immunosuppressant nonadherence in heart, liver, and lung transplant patients: associations with medication beliefs and illness perceptions. Transplantation.

[12] O'Grady JG, Asderakis A, Bradley R, Burnapp L, McPake DM, Perrin M, Russell S, Watson AR, Watson CJ, Wray J, Wilson LC (2010). Multidisciplinary insights into optimizing adherence after solid organ transplantation. Transplantation.

[13] Pew Research Center (2015). Device Ownership Over Time.

[14] McGillicuddy JW, Weiland AK, Frenzel RM, Mueller M, Brunner-Jackson BM, Taber DJ, Baliga PK, Treiber FA (2013). Patient attitudes toward mobile phone-based health monitoring: questionnaire study among kidney transplant recipients. J Med Internet Res.

[15] Suffoletto B, Calabria J, Ross A, Callaway C, Yealy DM (2012). A mobile phone text message program to measure oral antibiotic use and provide feedback on adherence to patients discharged from the emergency department. Acad Emerg Med.

[16] Bell AM, Fonda SJ, Walker MS, Schmidt V, Vigersky RA (2012). Mobile phone-based video messages for diabetes self-care support. J Diabetes Sci Technol.

[17] Strandbygaard U, Thomsen SF, Backer V (2010). A daily SMS reminder increases adherence to asthma treatment: a three-month follow-up study. Respir Med.

[18] Kunawararak P, Pongpanich S, Chantawong S, Pokaew P, Traisathit P, Srithanaviboonchai K, Plipat T (2011). Tuberculosis treatment with mobile-phone medication reminders in northern Thailand. Southeast Asian J Trop Med Public Health.

[19] Lester RT, Ritvo P, Mills EJ, Kariri A, Karanja S, Chung MH, Jack W, Habyarimana J, Sadatsafavi M, Najafzadeh M, Marra CA, Estambale B, Ngugi E, Ball TB, Thabane L, Gelmon LJ, Kimani J, Ackers M, Plummer FA (2010). Effects of a mobile phone short message service on antiretroviral treatment adherence in Kenya (WelTel Kenya1): a randomised trial. Lancet.

[20] Kim SI, Kim HS (2008). Effectiveness of mobile and internet intervention in patients with obese type 2 diabetes. Int J Med Inform.

[21] McGillicuddy JW, Gregoski MJ, Weiland AK, Rock RA, Brunner-Jackson BM, Patel SK, Thomas BS, Taber DJ, Chavin KD, Baliga PK, Treiber FA (2013). Mobile health medication adherence and blood pressure control in renal transplant recipients: a proof-of-concept randomized controlled trial. JMIR Res Protoc.

[22] Morgan D, Krueger R (1998). The Focus Group Kit.

[23] Chisholm-Burns M, Pinsky B, Parker G, Johnson P, Arcona S, Buzinec P, Chakravarti P, Good M, Cooper M (2012). Factors related to immunosuppressant medication adherence in renal transplant recipients. Clin Transplant.

[24] Butler JA, Peveler RC, Roderick P, Smith PW, Horne R, Mason JC (2004). Modifiable risk factors for non-adherence to immunosuppressants in renal transplant recipients: a cross-sectional study. Nephrol Dial Transplant.

[25] Schmid-Mohler G, Thut MP, Wüthrich RP, Denhaerynck K, De Geest S (2010). Non-adherence to immunosuppressive medication in renal transplant recipients within the scope of the Integrative Model of Behavioral Prediction: a cross-sectional study. Clin Transplant.

[26] Gordon EJ, Gallant M, Sehgal AR, Conti D, Siminoff LA (2009). Medication-taking among adult renal transplant recipients: barriers and strategies. Transpl Int.

[27] Evans RW, Applegate WH, Briscoe DM, Cohen DJ, Rorick CC, Murphy BT, Madsen JC (2010). Cost-related immunosuppressive medication nonadherence among kidney transplant recipients. Clin J Am Soc Nephrol.

[28] Griva K, Davenport A, Harrison M, Newman SP (2012). Non-adherence to immunosuppressive medications in kidney transplantation: intent vs. forgetfulness and clinical markers of medication intake. Ann Behav Med.

[29] Rosenstock IM, Strecher VJ, Becker MH (1988). Social learning theory and the Health Belief Model. Health Educ Q.

[30] Bandura A (1986). Social Foundations of Thought and Action: A Social Cognitive Theory.

[31] Thaler HR, Sunstein CR (2008). Nudge: Improving Decisions About Health, Wealth, and Happiness.

[32] Dayer L, Heldenbrand S, Anderson P, Gubbins PO, Martin BC (2013). Smartphone medication adherence apps: potential benefits to patients and providers: response to Aungst. J Am Pharm Assoc (2003).

[33] Goldade K, Sidhwani S, Patel S, Brendt L, Vigliaturo J, Kasiske B, Ahluwalia JS, Israni AK (2011). Kidney transplant patients' perceptions, beliefs, and barriers related to regular nephrology outpatient visits. Am J Kidney Dis.

[34] Patel MS, Asch DA, Volpp KG (2015). Wearable devices as facilitators, not drivers, of health behavior change. JAMA.

[35] Miloh T, Annunziato R, Arnon R, Warshaw J, Parkar S, Suchy FJ, Iyer K, Kerkar N (2009). Improved adherence and outcomes for pediatric liver transplant recipients by using text messaging. Pediatrics.

